# Precision estimation of winter wheat crop height and above-ground biomass using unmanned aerial vehicle imagery and oblique photoghraphy point cloud data

**DOI:** 10.3389/fpls.2024.1437350

**Published:** 2024-09-18

**Authors:** Yafeng Li, Changchun Li, Qian Cheng, Li Chen, Zongpeng Li, Weiguang Zhai, Bohan Mao, Zhen Chen

**Affiliations:** ^1^ School of Surveying and Land Information Engineering, Henan Polytechnic University, Jiaozuo, China; ^2^ Institute of Farmland Irrigation, Chinese Academy of Agricultural Sciences, Xinxiang, China; ^3^ Xingtai Academy of Agricultural Sciences, Xingtai, China

**Keywords:** unmanned aerial vehicle, vegetation indices, accumulated incremental height, crop height, above-ground biomass

## Abstract

**Introduction:**

Crop height and above-ground biomass (AGB) serve as crucial indicators for monitoring crop growth and estimating grain yield. Timely and accurate acquisition of wheat crop height and AGB data is paramount for guiding agricultural production. However, traditional data acquisition methods suffer from drawbacks such as time-consuming, laborious and destructive sampling.

**Methods:**

The current approach to estimating AGB using unmanned aerial vehicles (UAVs) remote sensing relies solely on spectral data, resulting in low accuracy in estimation. This method fails to address the ill-posed inverse problem of mapping from two-dimensional to three-dimensional and issues related to spectral saturation. To overcome these challenges, RGB and multispectral sensors mounted on UAVs were employed to acquire spectral image data. The five-directional oblique photography technique was utilized to construct the three-dimensional point cloud for extracting crop height.

**Results and Discussion:**

This study comparatively analyzed the potential of the mean method and the Accumulated Incremental Height (AIH) method in crop height extraction. Utilizing Vegetation Indices (VIs), AIH and their feature combinations, models including Random Forest Regression (RFR), eXtreme Gradient Boosting (XGBoost), Gradient Boosting Regression Trees (GBRT), Support Vector Regression (SVR) and Ridge Regression (RR) were constructed to estimate winter wheat AGB. The research results indicated that the AIH method performed well in crop height extraction, with minimal differences between 95% AIH and measured crop height values were observed across various growth stages of wheat, yielding R^2^ ranging from 0.768 to 0.784. Compared to individual features, the combination of multiple features significantly improved the model’s estimate accuracy. The incorporation of AIH features helps alleviate the effects of spectral saturation. Coupling VIs with AIH features, the model’s R^2^ increases from 0.694-0.885 with only VIs features to 0.728-0.925. In comparing the performance of five machine learning algorithms, it was discovered that models constructed based on decision trees were superior to other machine learning algorithms. Among them, the RFR algorithm performed optimally, with R^2^ ranging from 0.9 to 0.93.

**Conclusion:**

In conclusion, leveraging multi-source remote sensing data from UAVs with machine learning algorithms overcomes the limitations of traditional crop monitoring methods, offering a technological reference for precision agriculture management and decision-making.

## 1 Introduction

Wheat, as one of China’s three major cereal crops, plays a pivotal role in agricultural production. Given the impact of global population growth and climate change, monitoring the growth status of wheat and achieving stable growth in wheat production are imperative for realizing sustainable agricultural development, ensuring national food security and meeting market demands (Zhu et al., 2023a). Crop height and above-ground biomass (AGB) are considered crucial indicators reflecting the wheat growth status and essential components of grain yield. Real-time and accurate estimation of winter wheat crop height and AGB at various growth stages is paramount importance for guiding fertilizer application, managing irrigation, estimating crop yield and informing national macro-level decision-making (Huang et al., 2016).

Traditional methods for collecting AGB typically involve the destructive acquisition of crops in the field. While the measurement results are relatively accurate, there are drawbacks such as time-consuming, laborious, elevated costs and poor timeliness. At the same time, the random sampling in the manual data collection process lacks spatial variability, which may result in data that does not adequately represent the different characteristics and conditions within the small planting area (Meng et al., 2016). In recent years, remote sensing as a novel technological approach, has provided a fresh alternative for estimating AGB in crops, owing to its non-contact, convenient, efficient and flexible characteristics. Remote sensing technology, with its ability to capture the spectral reflectance characteristics of vegetation, offers extensive and high spatiotemporal resolution surface information. Therefore, it demonstrates significant potential in various studies such as crop growth monitoring, pest and disease prediction (Pinto et al., 2020), AGB estimation (Yue et al., 2019), yield and quality prediction (Maimaitiyiming et al., 2019; Chen, 2020). The large-scale estimation of AGB based on satellite remote sensing has been widely applied in the fields of forests and grasslands (Chen, 2015; Wang et al., 2019). However, for the retrieval of AGB in small-scale agricultural fields, higher spatial and spectral resolution is required. In recent years, unmanned aerial vehicle (UAV) remote sensing technology has rapidly developed, providing the possibility for precise monitoring and large-scale applications due to its advantages of high resolution, flexibility, and low cost (Sagan et al., 2019). Currently, UAV remote sensing employs various sensors for crop phenotyping analysis, including RGB, multispectral, hyperspectral and Light Detection and Ranging (LiDAR) sensors. Hyperspectral sensors offer detailed spectral information but widespread adoption are hindered by high costs and complexity. On the contrary, multispectral sensors are affordable, easy to handle and encompass several key bands commonly used for crop growth monitoring (Kross et al., 2015). Compared to hyperspectral sensors, multispectral sensors are more widely used in crop phenotyping analysis (Feng et al., 2020). The single-band reflectance of a spectrum provides information only about a specific wavelength range, which may limit a comprehensive understanding of crop canopy features. In contrast, vegetation indices are simple mathematical combinations or transformations of reflectance in two or more spectral channels to represent vegetation status conditions, capable of highlighting specific features or details of crops. Several vegetation indices are widely used to assess information such as the growth status, coverage, biomass and productivity of crop (Wei et al., 2021). Wang et al. utilized multispectral images to construct 44 vegetation indices and employed three machine learning algorithms to predict the AGB of winter wheat (Wang et al., 2022). Despite biomass estimation based on spectral features being a hot topic in remote sensing crop phenotyping analysis, spectral saturation is a common issue during the later stages of crop growth. This phenomenon often leads to lower accuracy in biomass estimation models (Fu et al., 2014). To address such issues, previous studies have tackled the overreliance on spectral data in inversion models by incorporating texture features (Xu et al., 2022), canopy coverage (Lee and Lee, 2013) and elevation data (Tilly et al., 2015). By incorporating these additional features, the accuracy of inversion models is improved, and stability is enhanced.

Crop height and AGB are crucial indicators of crop growth status. Previous studies have found that integrating crop height has a positive impact on improving the accuracy of AGB estimation and addressing spectral saturation issues (Malambo et al., 2018). With the advancement of remote sensing technology, the methods for measuring crop height have shifted from traditional manual approaches to extraction utilizing UAV images. However, nadir photography often captures only limited canopy information, lacking highly accurate crop height details. Utilizing UAVs equipped with visible light cameras for oblique photogrammety offers significant advantages, including the capture of color information, higher point cloud density in the horizontal direction, lower cost and greater flexibility. Methods for measuring crop height values using the UAV-mounted RGB cameras primarily involve the mean method and the accumulated incremental height (AIH) method. In a statistical unit, the AIH is calculated by sorting all normalized point clouds by height and computing the accumulated height of all points. The accumulated height percentile of X% of the points within each statistical unit represents the percentile of accumulated height for that unit. Li et al. established a crop height retrieval model based on the mean crop height and the 50% and 90% AIH, comparing with field measurements (Li et al., 2016). They found that mean height value and the 90% AIH can effectively represent crop height in the crop height model. Jimenez-Berni et al. utilized point cloud data to construct various AIH and found that the 95.5% AIH exhibited the smallest error, demonstrating a strong correlation with the true values (Jimenez-Berni et al., 2018). Therefore, due to the high degree of alignment between the predicted values obtained based on the accumulated incremental height method and the actual values. It is anticipated that coupling AIH and spectral features will further enhance the accuracy of estimating crop phenotypic parameters. Previous studies have already confirmed the correlation between height indicators and crop biomass (Madec et al., 2017). Crop height indicators, such as AIH, obtained from drone imagery, have been identified as key variables for estimating crop biomass (Li et al., 2016; Kotivuori et al., 2020; Lu et al., 2021).

With the advancement of digital photogrammetry, multi-view stereo vision technology and other advanced techniques, it has become feasible to reconstruct the 3-Dimension (3D) point cloud based on multi-view images (Jayathunga et al., 2018). Building upon the acquisition of two-dimensional images through oblique photography, the structure from motion (SFM) algorithm autonomously seeks and connects matching points to derive relative depth information in three-dimensional space, establishing a high precision 3D point cloud. Currently, one of the most widely used methods for obtaining crop height is to utilize the UAV equipped with RGB camera to perform three-dimensional reconstruction of images and generate point cloud. The canopy structure information derived from point clouds generated through oblique photography has found extensive application in estimating tree biomass (Wallace et al., 2017). Lu et al. combined vegetation indices (VIs) with crop height to enhance the accuracy of wheat AGB prediction (Lu et al., 2019). Maimaitijiang et al. captured RGB images and employed oblique photography to construct point cloud, assessed the potential of the vegetation index weighted canopy volume model (CVMVI), which integrates canopy spectral and volume information, in estimating AGB for soybeans (Maimaitijiang et al., 2019).

With the increasing maturity of artificial intelligence, machine learning, and other algorithms, various machine learning algorithms have been extensively applied in crop monitoring (Singh et al., 2023). By coupling remote sensing data from different periods with machine learning algorithms, it is possible to more accurately reveal the growth patterns of winter wheat. Ridge regression (RR) is suitable for handling the linear relationship between remote sensing variables and crop biochemical parameters, aiming to enhance the stability of the model. In comparison to linear regression algorithms, non-linear regression algorithms such as Random Forest Regression (RFR), Support Vector Regression (SVR), eXtreme Gradient Boosting (XGBoost), and Gradient Boosting Regression Trees (GBRT) can handle high-dimensional data and non-linear relationships. Studies have demonstrated that machine learning regression algorithms exhibited higher accuracy in biomass estimation compared to traditional regression algorithms, yielding superior regression results (Li et al., 2016). The RFR algorithm, initially proposed by Leo Breiman, Adele Cutler and others, falls under the ensemble learning method Bagging and is applicable to both classification and regression tasks. By combining multiple weak classifiers, the model achieves high accuracy and generalization performance (Ehlers et al., 2022). GBRT is a type of ensemble learning, specifically belonging to Boosting. It comprises Regression Trees (RT) and Gradient Boosting (GB). It is an iterative ensemble algorithm for regression decision trees. It uses gradient descent to iterate over new learners. The core idea is that each tree learns the conclusions and residuals of all the previous trees. The objective is to minimize the difference between the true values and predicted values, and the conclusions of all regression trees are accumulated to obtain the final result (Wen et al., 2022). XGBoost is an abbreviation for eXtreme Gradient Boosting, which is a decision tree ensemble regression algorithm that combines base functions with weights to enhance data fitting. It demonstrates increased efficiency when dealing with large-scale datasets and complex models. The algorithm follows the gradient boosting approach by iteratively training a series of weak learners (typically decision trees) to correct the residuals from the previous iteration. Through iterations, it continually enhances the overall performance of the model, ultimately combining these weak learners into a strong learner. In comparison to the GBRT algorithm, XGBoost introduces regularization terms in the loss function (L1: alpha, L2: lambda). As some loss functions pose challenges in computing derivatives, XGBoost utilizes the second-order Taylor expansion of the loss function as a fitting, which helps mitigate the impact of overfitting (Zhang et al., 2022). SVR is founded on the vapnik-chervonenkis dimension theory and the principle of structural risk minimization. It addresses the challenge of function approximation and is grounded in ordered risk minimization, forming a small-sample statistical theory. SVR exhibits the capability to alleviate overfitting to some extent, providing good stability and generality. It has found widespread applications in various fields such as computer vision, data analysis and mining (Abdollahpour et al., 2020; Li et al., 2021). Ridge Regression is a regression method employed for the analysis of collinear data, offering biased estimates to tackle issues such as multicollinearity in regression analysis. It essentially serves as an improved method of the ordinary least squares estimation, aiming to alleviate the adverse effects of multicollinearity. The concept behind RR is that when multicollinearity is present in the data, a small ridge parameter k (0<k<1) is introduced and added to the main diagonal elements of the matrix x'x . This adjustment makes the degree of singularity approached by x'x+k is much smaller than the degree of singularity approached by x'x , leading to enhanced stability in parameter estimation (Li et al., 2023). Zhang et al. utilized Landsat imagery along with five machine learning algorithms, namely SVR, RFR, k-Nearest Neighbors (k-NN) and Artificial Neural Network (ANN), for predicting grass biomass (Zhang et al., 2018). The research results indicated that ANN and SVR produced similar outcomes in estimating biomass. Simultaneously, the stability of different algorithms is significantly affected by the number of features. An excessive number of features can lead to overfitting and the curse of dimensionality, while a small number of features may easily result in underfitting. Therefore, conducting correlation analysis or feature selection can enhance the accuracy and stability of the algorithms (Zhai et al., 2023).

While the inversion of crop physiological parameters based on UAV RGB and multispectral images has been extensively utilized (Yue et al., 2019; Sun et al., 2021), there is limited research on integrating crop height indicators and spectral information provided by UAV remote sensing with machine learning to estimate winter wheat AGB. Additionally, relying solely on spectral information for AGB inversion is susceptible to spectral saturation phenomena. In summary, the primary objectives of this study are as follows: (1) To quantify the potential of the mean method and AIH method in extracting wheat crop height. (2) To analyze the impact of AIH, VIs and their feature combinations on AGB estimation at different growth stages of wheat. (3) To explore the performance of different machine learning algorithms in winter wheat AGB estimation, providing references and support for precision agricultural management.

## 2 Materials and methods

### 2.1 Study area and experimental design

**Figure 1:**
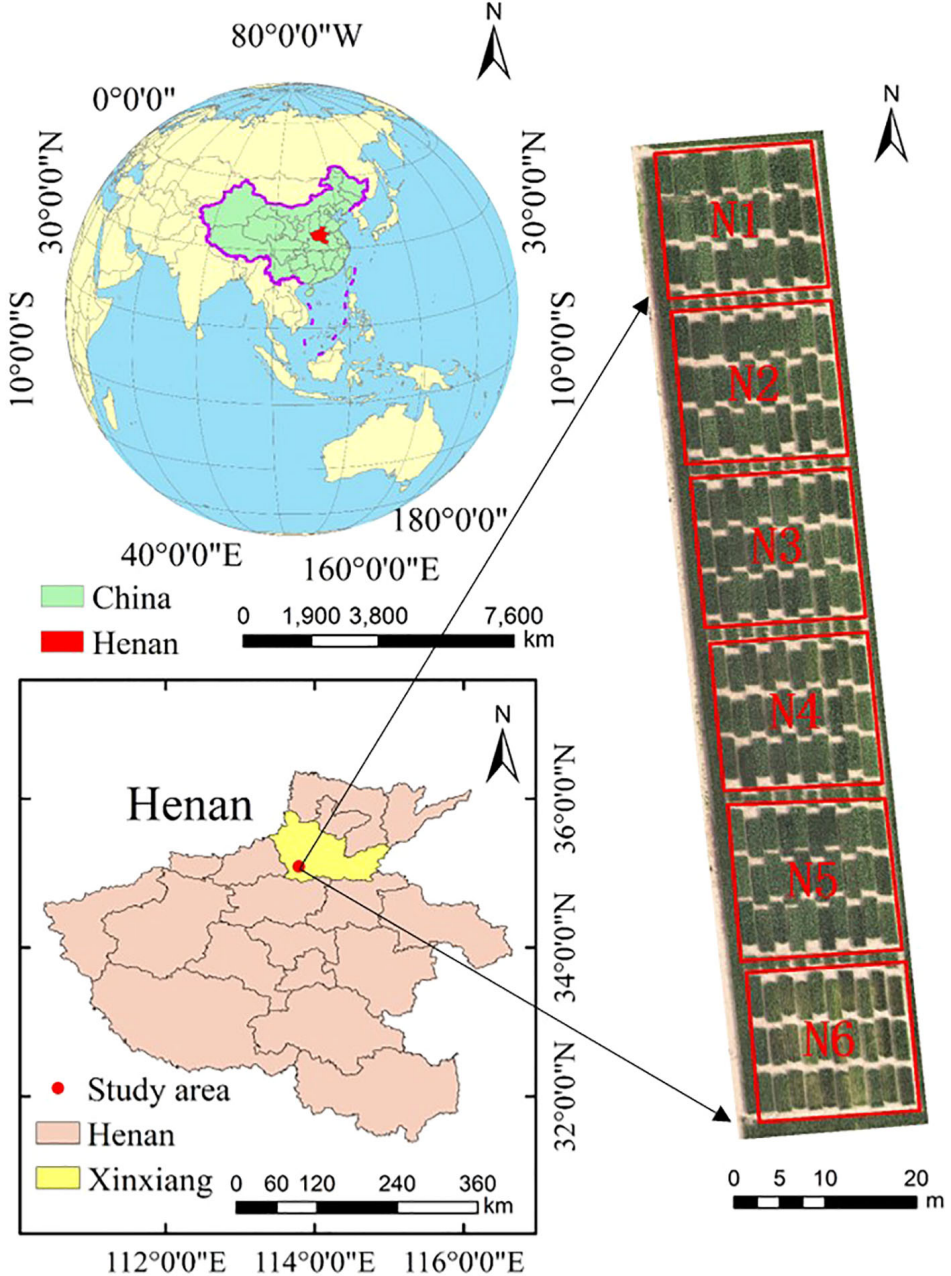
Location and experimental design of the study area. The red boxes represent different treatment.

The study area is situated at the Xinxiang Comprehensive Experimental Base of the Chinese Academy of Agricultural Sciences (35.2°N, 113.8°E), with an annual average temperature of 14°C and precipitation of approximately 573.4 mm, it was suitable for the growth of winter wheat. Daily irrigation was carried out using moving lateral irrigation machines. The experiment designed six different nitrogen fertilizer treatments (N1: 300 kg/ha, N2: 240 kg/ha, N3: 180 kg/ha, N4: 120 kg/ha, N5: 60 kg/ha, N6: 0 kg/ha). Each treatment encompassed 30 plots, totaling 180 plots. Each plot measured 1.4m×4m, with a planting row spacing of 15 cm, and the basic seedling density was around 150,000 plants per mu, as illustrated in **Figure 1**. Nitrogen fertilizer was administered to wheat during the jointing and heading stage, with a 2:1 ratio for fertilizer distribution. Other field management practices adhered to the local conditions of winter wheat production (Yang et al., 2020). To ensure data quality, a total of 21 ground control points were set up in the study area, and precise coordinates for these control points were obtained using Global Navigation Satellite System technology.

### 2.2 Data acquisition

#### 2.2.1 Field data acquisition

Since the jointing stage, heading stage and grain filling stage represent key transition points in wheat growth, they can comprehensively reflect the growth status and yield potential of wheat at different developmental stages. This study conducted experiments during the jointing stage, heading stage and grain filling stages, primarily collecting data on the crop height and AGB of winter wheat. The crop height was measured using a ruler with millimeter precision. Six random measurements were taken within each experimental plot, and the average value was used as the true height of winter wheat. For above-ground biomass assessment, six representative wheat plants were selected as samples in each plot. After measuring the fresh weight, the samples were placed in paper bags and baked at 105°C in a drying oven for 30 minutes. Subsequently, the temperature was adjusted to 75°C, and the samples were baked until a constant weight was reached (approximately 24-48 hours). After the dry weight of each sample was determined, the winter wheat AGB (t/hm²) per unit area was calculated based on population density and sample dry weight. **Table 1** summarizes the statistical information of winter wheat crop height and AGB parameters.

**Table 1:** Statistics of crop height and AGB parameters at different growth stages.

Growth stages	Sample size	Crop Height(m)				AGB(t/hm^2^)			
		Min	Max	Mean	CV(%)	Min	Max	Mean	CV(%)
Jointing stage	180	0.362	0.588	0.484	8.74	0.16	3.92	2.33	37.72
Heading stage	180	0.635	0.913	0.768	7.27	2.18	7.012	4.96	21.67
Grain filling stage	180	0.660	1.012	0.836	8.08	4.48	10.80	8.25	15.56

CV, coefficient of variation, Used to describe the central tendency and dispersion of data.

#### 2.2.2 Acquisition and preprocessing of UAV remote sensing data

**Figure 2:**
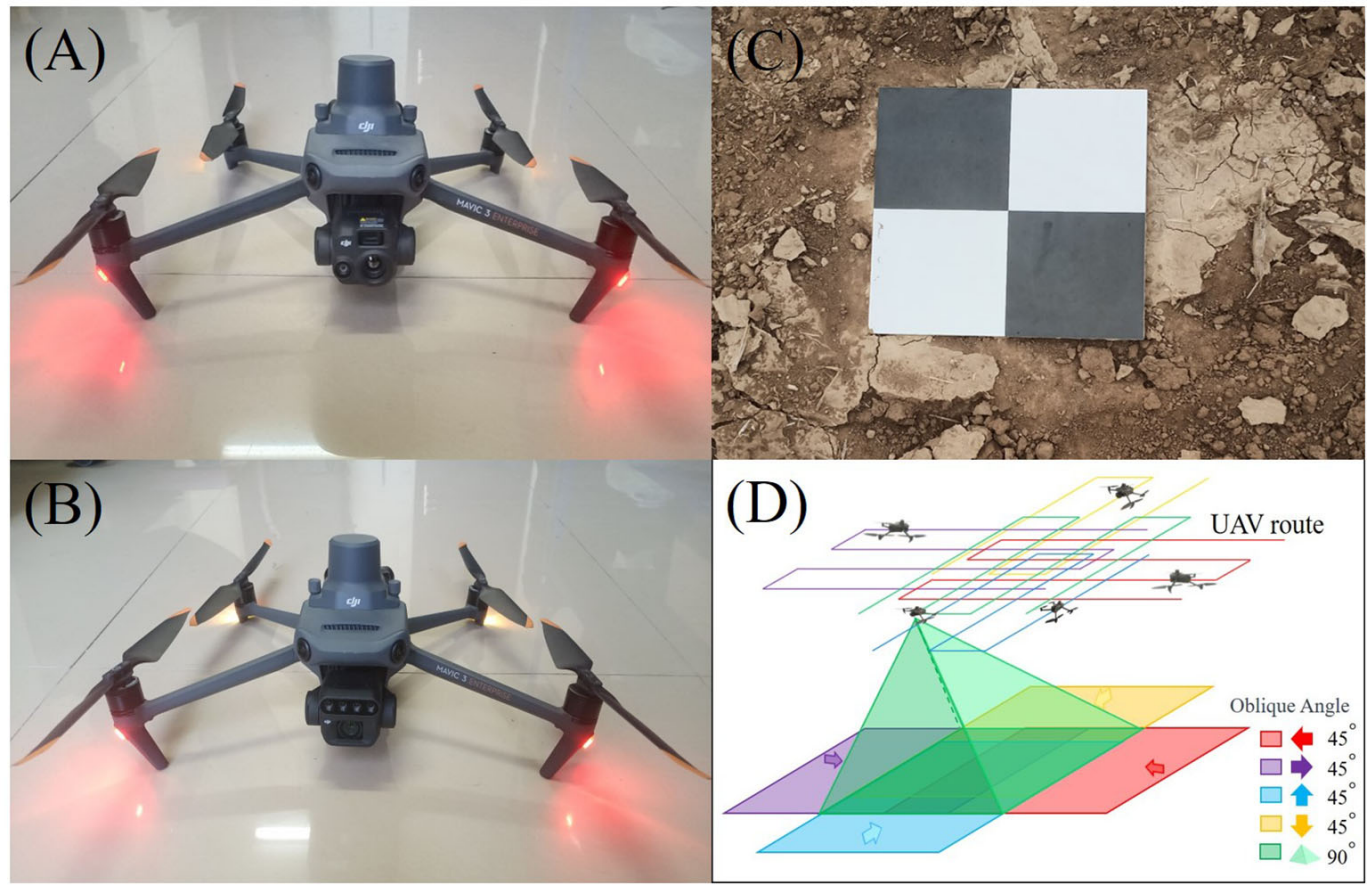
The UAV and working principles. **(A)** DJI Mavic 3T. **(B)** DJI Mavic 3M. **(C)** ground control points. **(D)** oblique photography principle.

In this study, DJI Mavic 3M and DJI Mavic 3T (SZ DJI Technology Co., Shenzhen, China) were utilized for UAV data acquisition (**Figure 2**). To mitigate the impact of changes in solar zenith angle, images were collected between 11:30 a.m. and 12:30 p.m. on sunny days. The RGB sensor on the DJI Mavic 3M had 20 MP effective pixels and 24 mm format equivalent. The multispectral sensor had 5 MP effective pixels and 25 mm format equivalent, and a total of four bands: green (G, 560 ± 16nm), red (R, 650 ± 16nm), red edge (RE, 730 ± 16nm) and near infrared (NIR, 860 ± 26nm). For capturing RGB and multispectral images in the study area, DJI Mavic 3M was utilized for the flight. The flight routes were planned using DJI Pilot 2 (SZ DJI Technology Co., Shenzhen, China), with a photography mode set to time interval shot. The UAV’s camera was maintained vertical to the ground, and the relative flying height was set to 30 meters. The forward overlap rate and the side overlap rate were both set at 80%. Oblique photography data were collected using the RGB sensor equipped on DJI Mavic 3T. The RGB sensor had 48 MP effective pixels and 24 mm format equivalent. To generate point cloud data for winter wheat through 3D reconstruction, a five-way oblique photography mode with a tilt angle of 45 degrees (vertical downward, forward oblique, backward oblique, left oblique and right oblique) was employed. To determine the bare ground height, images of the bare soil were immediately captured after the completion of wheat planting.

After completing the UAV’s flight mission, the RGB and multispectral images were aligned and stitched using Pix4Dmapper 4.5.6 (Pix4D, Lausanne, Switzerland), resulting in the generation of orthomosaic. The processing workflow included key steps such as importing ground control points (GCPs), georeferencing, image alignment, building dense point clouds and radiometric correction. Subsequently, utilizing ArcGIS 10.6 (Environmental Systems Research Institute, Inc., Redlands, CA, USA), georeferencing was applied to UAV images taken at various growth stages, and vegetation index maps were generated. VIs were extracted by drawing polygon vectors for each plot. Building upon an analysis of existing research literature, this study extracted 18 vegetation index features based on the spectral information from RGB and multispectral imagery, as shown in **Table 2**.

**Table 2:** Vegetation indices used in this study.

Vegetation Indices	Formula	Reference
Chlorophyll Index-Red Edge (CI-REG)	(NIR/EDGE)−1	(Gitelson et al., 2003)
Two-Band Enhanced Vegetation Index (EVI2)	(2.5(NIR−R))/(1+NIR+2.4R)	(Jiang et al., 2008)
Excess Green Index (ExG)	2G−R−B	(Zhang et al., 2019)
ExG-ExR Vegetation Index (ExGR)	(2G−R−B)−(1.4R−G)	(Meyer and Neto, 2008)
Green Leaf Index (GLI)	(2G−R−B)/(2G+R+B)	(Zhang et al., 2019)
Kernel Normalized Difference Vegetation Index (kNDVI)	$\tanh{\left((\text{NIR}-\text{R})/\left(2\times \sigma \right)\right)}^{2}$	(Camps-Valls et al., 2021)
Leaf Chlorophyll Index (LCI)	(NIR−EDGE)/(NIR−R)	(Xiao et al., 2014)
Normalized Difference Vegetation Index (NDVI)	(NIR−R)/(NIR+R)	(Li et al., 2004)
Normalized Green Red Difference Index (NGRDI)	(G−R)/(G+R)	(Li et al., 2019)
Optimized Soil-Adjusted Vegetation Index (OSAVI)	1.6[(NIR−R)/(NIR+R+0.16)]	(Blanco et al., 2020)
Renormalized Difference Vegetation Index (RDVI)	$(\text{NIR}-\text{R})/\sqrt{\left(\text{NIR}+\text{R}\right)}$	(Roujean and Breon, 1995)
Renormalized Difference Vegetation Index - Red Edge (RDVI-REG)	$(\text{NIR}-\text{EDGE})/\sqrt{\left(\text{NIR}+\text{EDGE}\right)}$	(Roujean and Breon, 1995)
Red Green Blue Vegetation Index (RGBVI)	$\left({\text{G}}^{2}-(\text{R}\times \text{B}))/({\text{G}}^{2}+(\text{R}\times \text{B})\right)$	(Bendig et al., 2015)
Core Red Edge Triangular Vegetation Index (RTVI-CORE)	100(NIR−EDGE)−10(NIR−G)	(Walsh et al., 2018)
Ratio Vegetation Index (RVI)	NIR/R	(Liu et al., 2021)
Visible Atmospherically Resistant Index (VARI)	(G−R)/(G+R+B)	(Huang et al., 2023)
visible-band difference vegetation index (VDVI)	(2G−(R+B))/(2G+(R+B))	(Li et al., 2022)
Vegetative Index (VEG)	$\;\text{G}/\left({\text{R}}^{\alpha }{\text{B}}^{1-\alpha }\right)\;$	(Hague et al., 2006)

R, red band reflectivity; G, green band reflectivity; B, blue band reflectivity; NIR, near infrared band reflectivity; EDGE, red edge band reflectivity. α=0.667, σ=0.5(NIR+RED).

#### 2.2.3 Crop height extraction

**Figure 3:**
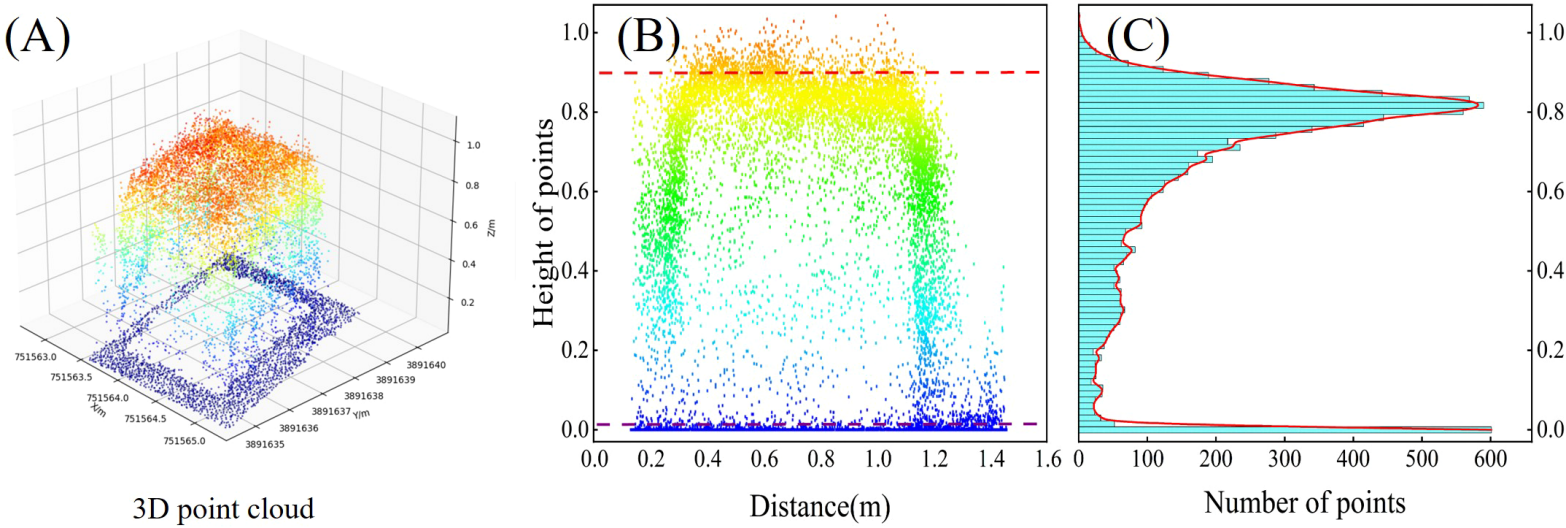
Crop height variables. **(A)** Point cloud distribution in a single plot. **(B)** Profile point cloud in a single plot (the red line represents 95% AIH, and the purple line represents ground height). **(C)** Number of point clouds at different heights in the profile.

Utilizing DJI Terra software (SZ DJI Technology Co., Shenzhen, China) for three-dimensional reconstruction of images obtained through oblique photography, the reconstructed point cloud data was imported into LiDAR360 (V. 5.2, Green Valley, Co. Ltd. Beijing, China) for preprocessing. This preprocessing encompassed essential tasks, including clipping, denoising, filtering and normalization of the point cloud data. Based on the normalized point cloud data, the point cloud was sorted in ascending order of height to generate the AIH. The Canopy Height Model (CHM) was commonly used for estimating crop height. Digital Surface Model (DSM) with a resolution of 0.5 meters and Digital Elevation Model (DEM) were generated through Kriging interpolation. CHM was obtained by subtracting DSM from DEM. For each research plot, the mean estimated crop height was computed by summing all pixel values in the CHM and dividing by the total number of pixels. The AIH method selected the height value at a lower AIH as the baseline and the height value at a higher AIH as the upper boundary of the vegetation. The difference between these two height values yielded the estimated crop height for the study area. In this study, 99%, 95%, 90% and 80% AIH were individually used for crop height prediction, aiming to identify the optimal AIH. To accurately predict wheat AGB and explore the impact of AIH features on AGB estimation, this study evaluated and analyzed the model’s estimation performance using extracted 5%, 20%, 40%, 60%, 80% and 95% AIH features. The crop point cloud distribution for the planting plot is shown in **Figure 3**.

### 2.3 Methods

#### 2.3.1 Machine learning algorithms

Machine learning regression algorithms have the capability to handle both linear and nonlinear relationships between remote sensing variables and crop biochemical parameters. Compared to other regression algorithms, machine learning algorithms exhibit superior performance in regression predictions that involve multiple input variables. This study developed wheat aboveground biomass (AGB) estimation models using five machine learning algorithms: RFR, GBRT, XGBoost, SVR and RR.

**Figure 4:**
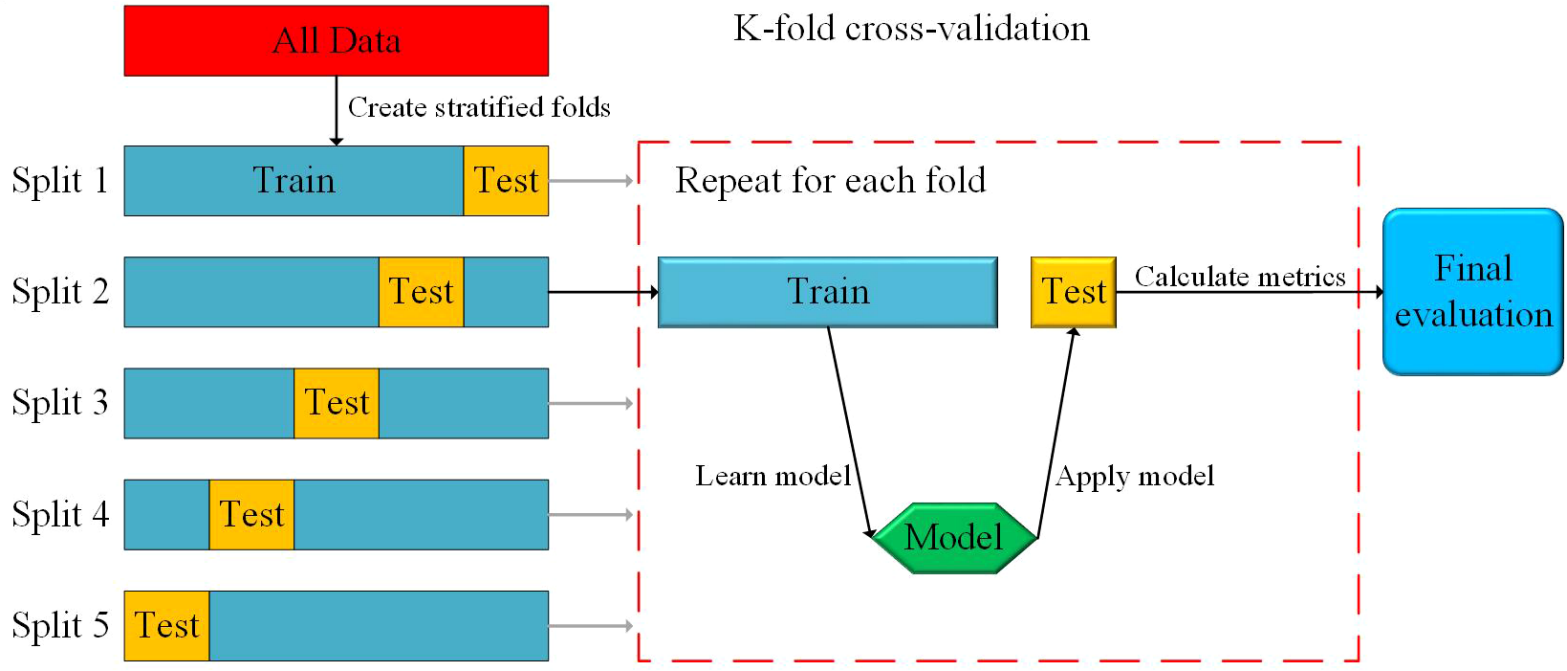
Overview of k-fold (5-fold) cross-validation for model evaluation.

K-fold cross-validation is a statistical method that divides data samples into smaller subsets (**Figure 4**). It can be employed as a method for accuracy testing and hyperparameter selection when dealing with small sample sizes (Shah et al., 2019).In this study, the data were divided into five folds through five-fold cross-validation. During each iteration, four folds are sequentially used as the training set, while the remaining fold serves as the validation dataset. Through five iterations, the model performance could be effectively evaluated. This approach was beneficial for improving overfitting and underfitting. The experiment encompassed 180 samples, including canopy feature data collected by different sensors and winter wheat AGB. These samples were divided into a training set and a test set in a 7:3 ratio. To ensure each model achieved optimal biomass prediction performance, the grid search algorithm was employed to determine the optimal parameters. The parameters set for the grid search algorithm include the model to be optimized, a dictionary of hyperparameter combinations for the model, 5-fold cross-validation, and the evaluation metric of root mean square error. Iterating through all parameter combinations, the optimal set of parameters for the highest accuracy model was determined. The RFR, GBRT, XGBoost, SVR and RR models, optimized with parameters, were utilized to predict the biomass of winter wheat at different growth stages in the region.

#### 2.3.2 Evaluation metrics

The model accuracy is evaluated using three metrics: R^2^, root mean square error (RMSE) and normalized root mean square error (nRMSE). The R^2^ value ranges from [0,1], with a higher value indicating better estimation performance of the model. Smaller values for RMSE and nRMSE improved predictive performance of the model.


(1) $$\begin{array}{c} R^{2}=1-\frac{\sum_{i=1}^{n}{\left(x_{i}-y_{i}\right)}^{2}}{\sum_{i=1}^{n}{\left(x_{i}-\bar{y}\right)}^{2}} \end{array}$$

(2) $$RMSE=\sqrt{\frac{\sum_{i=1}^{n}{\left(x_{i}-y_{i}\right)}^{2}}{n}}$$

(3) $$\begin{array}{c} \text{n}RMSE=\frac{RMSE}{\bar{y}}\times 100\% \end{array}$$

**Figure 5:**
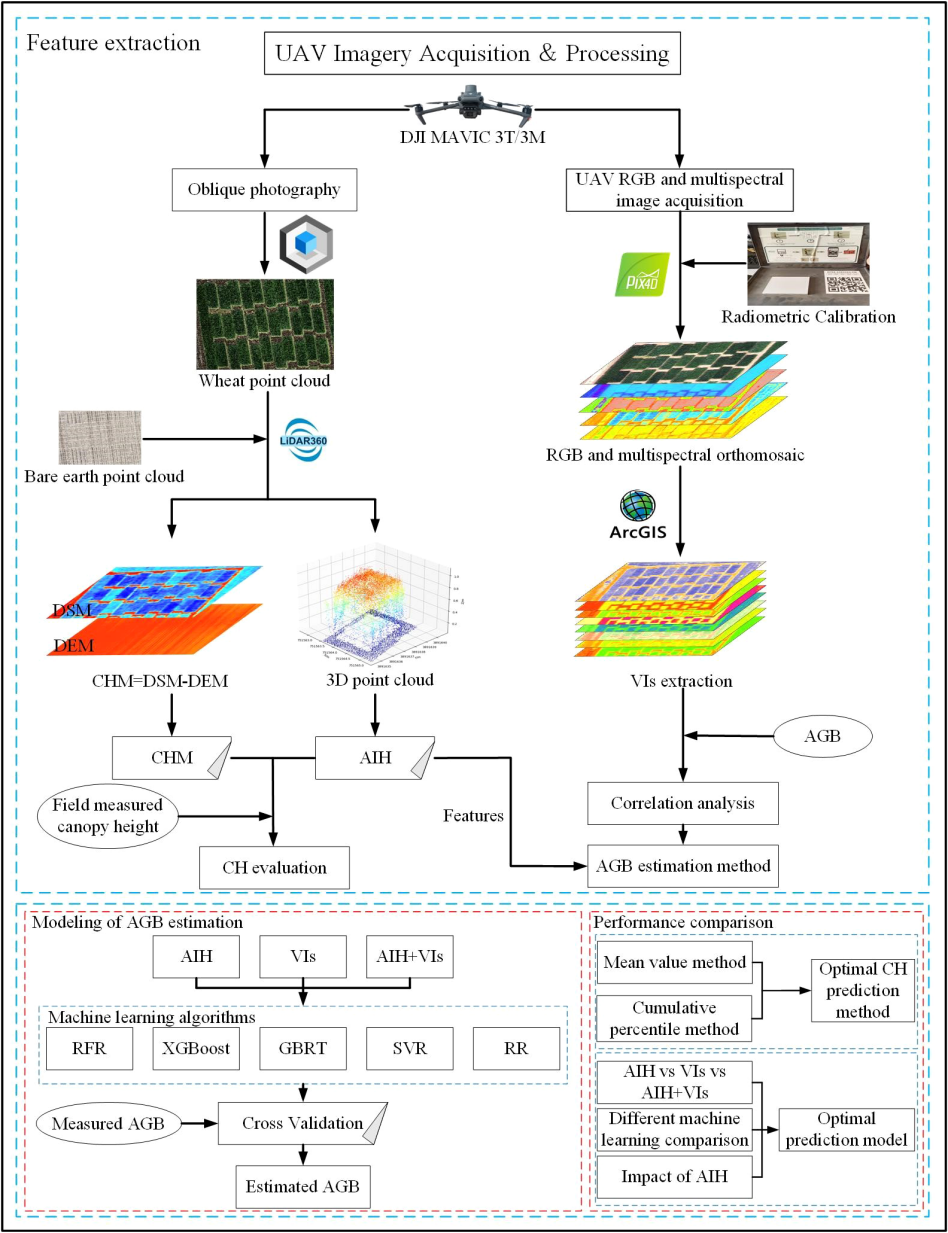
Experimental flowchart. (UAV, Unmanned Aerial Vehicle; DSM, Digital Surface Model; DEM, Digital Elevation Model; CHM, Canopy Height Model; CH, Crop Height; AIH, Accumulated Incremental Height; AGB, Above-Ground Biomass; VIs, Vegetation Indices; RFR, Random Forest Regression; XGBoost, eXtreme Gradient Boosting; GBRT, Gradient Boosting Regression Trees; SVR, Support Vector Regression; RR, Ridge Regression).

In the formula, $x_{i}\;$ represents the measured value of AGB; $y_{i}\;$ represents the estimated value of AGB; *n* is the number of samples; $\;\bar{y\;}$ is the average value of the measured values. In order to understand the experiment more intuitively, the experiment flowchart (**Figure 5**) was created based on the experiment design, data collection and processing.

## 3 Results

### 3.1 Winter wheat crop height estimation

**Figure 6:**
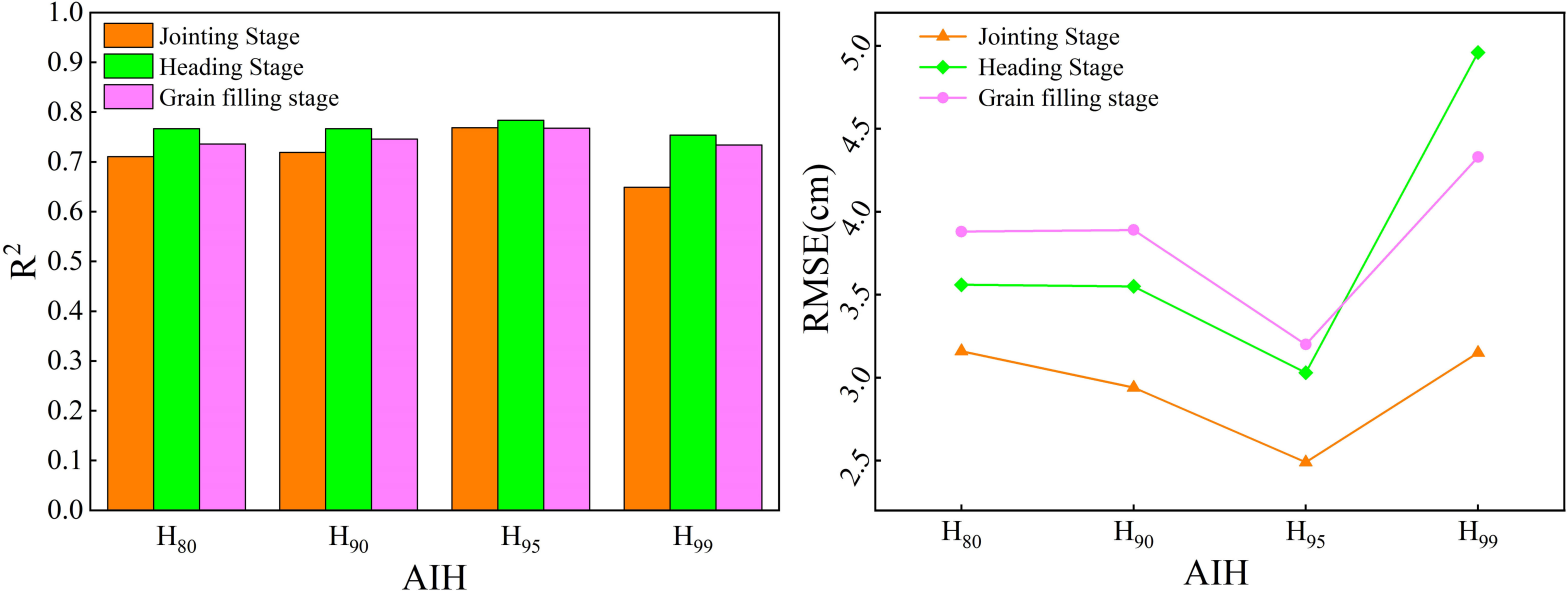
Performance of different AIH in estimating crop height.

**Figure 7:**
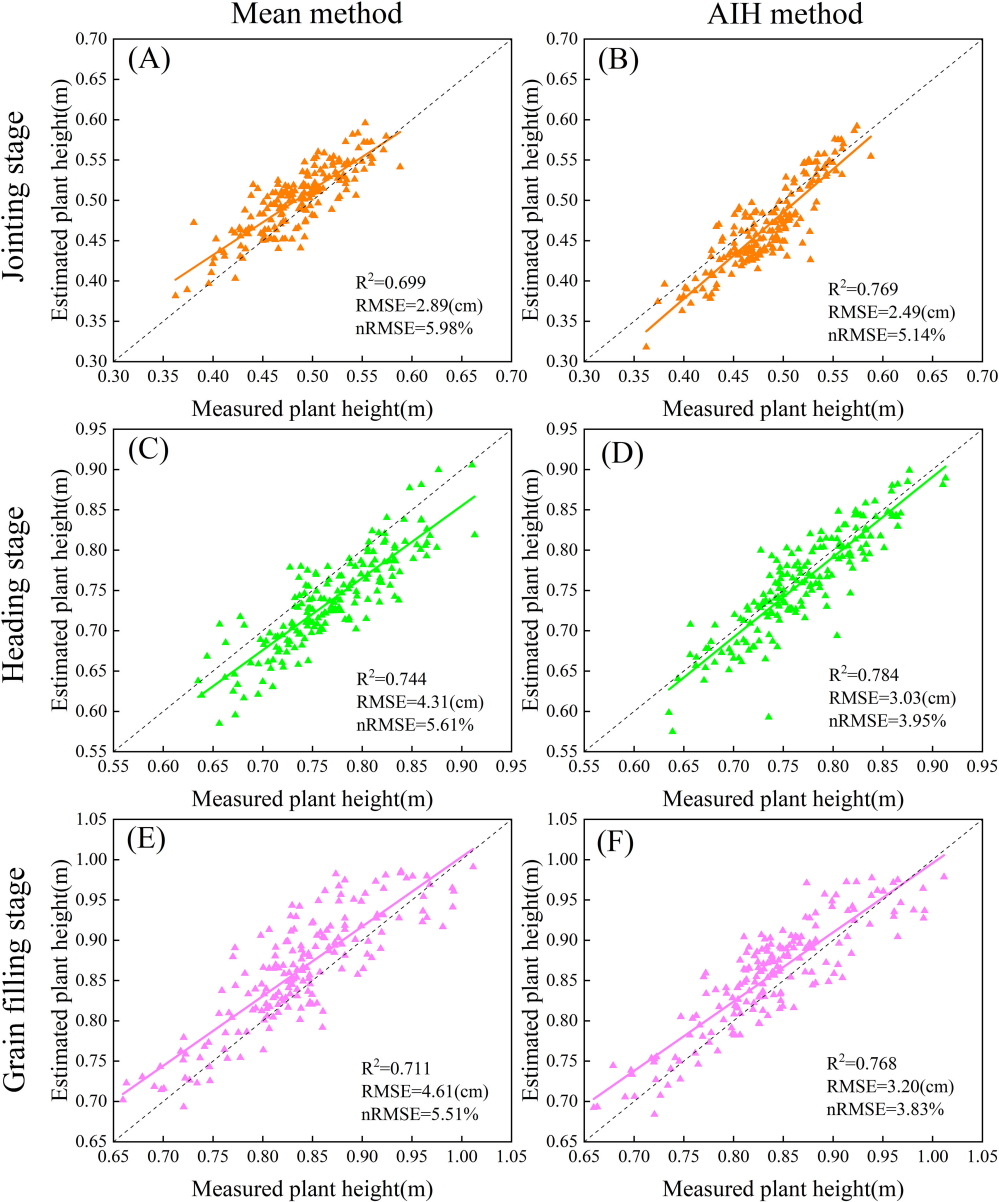
Scatter plot of estimated crop height based on CHM and 95% AIH. The first column displays variables for crop height estimation based on CHM, and the second column displays variables based on 95% AIH estimation. **(A, B)** Jointing stage. **(C, D)** Heading stage. **(E, F)** Grain filling stage. The dashed lines indicate the expected 1:1 relationship.

This study conducted the estimation of winter wheat crop height using both the mean method and the AIH method. To determine the optimal AIH for estimating crop height, crop height estimation was conducted using 80%, 90%, 95% and 99% AIH. **Figure 6** shows that the 95% AIH had the smallest error, with R^2^ ranging from 0.768-0.784. **Figure 7** illustrated the regression plots for crop height estimation during the jointing, heading and grain filling stages of winter wheat. It was observed that both methods exhibited good accuracy across different growth stages. The R^2^, RMSE and nRMSE values during the different growth stages follow similar changing trends, with ranges of 0.699-0.784, 2.49cm-4.61cm and 3.83%-5.98%, respectively. In the crop height extraction based on the mean method, the heading stage boasted the highest R^2^, with a value of 0.744, followed by the jointing and grain filling stages. The RMSE reached the minimum during the jointing stage, at 2.89 cm, while the nRMSE achieved the minimum during the grain filling stage, at 5.51%. In contrast, in the crop height extraction based on the 95% AIH, R^2^ reached its maximum during the heading stage, at 0.784. The RMSE attained its minimum during the jointing stage, at 2.49 m, which was relatively small. The nRMSE reached its minimum during the grain filling stage. Compared to the mean method, the AIH method exhibited higher R^2^ in all growth stages. The difference in R^2^ was particularly notable during the jointing stage, with a 7% gap, while the differences in the heading and grain filling stages were relatively small. Meanwhile, the RMSE and nRMSE values were slightly lower than those for the mean method. The RMSE and nRMSE showed the greatest variation during the grain filling stage, with changes of 1.41 cm and 1.68%. During the jointing and heading stages, changes were relatively small. In summary, compared to the mean method, the AIH method selected in this study for estimating winter wheat crop height demonstrated higher accuracy.

### 3.2 Estimation of AGB based on different features

**Figure 8:**
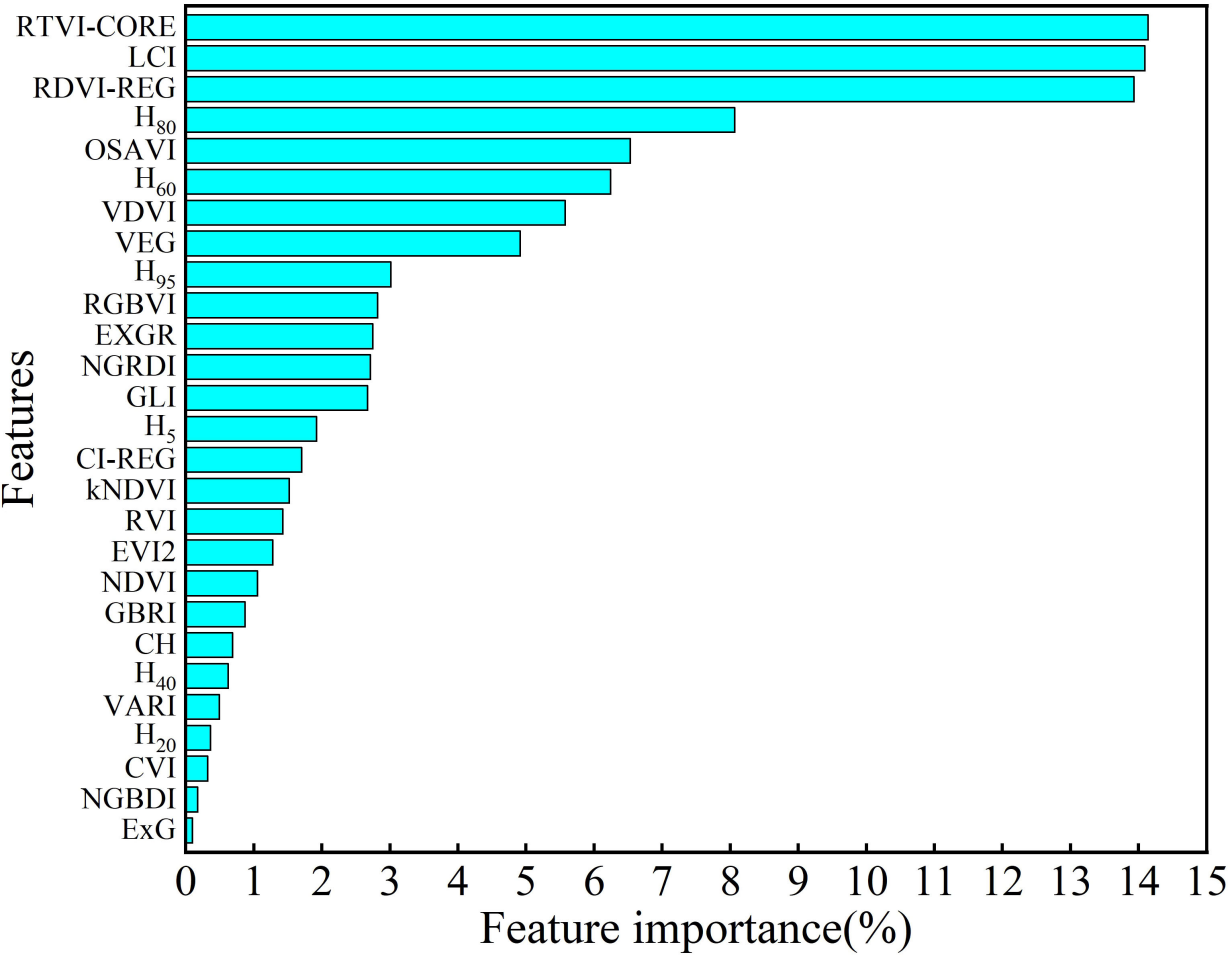
Feature importance analysis.

In order to prevent overfitting during the model training process and reduce the number of feature variables, this study utilized the feature importance analysis algorithm integrated into RFR. Feature importance analysis was conducted for both VIs and AIH features, as illustrated in **Figure 8**. RTVI-CORE, LCI, RDVI-REG, H_80_, OSAVI, and H_60_ exhibited relatively high feature importance, all exceeding 6%, with RTVI-CORE having the highest feature importance at 14.14%. Among the AIH features, except for H_20_ and H_40_, the other AIHs demonstrated relatively high feature importance. ExG, NGBDI, CVI, H_20_, VARI, H_40_, CH and GBRI had feature importance below 1%. Therefore, to prevent model overfitting, features such as ExG, NGBDI, GBRI, CH and H_40_ were excluded.

The estimation results for wheat AGB using AIH features, VIs features and their combinations were presented in **Table 3**. When predicting wheat AGB solely based on AIH features, the accuracy was relatively low, with R^2^ ranging from 0.416 to 0.632, RMSE ranging from 0.523 to 0.885 t/hm^2^ and nRMSE ranging from 9.11% to 31.23%. Estimating AGB based on VIs features showed better performance compared to using only AIH features, with R^2^ ranging from 0.694 to 0.885, RMSE ranging from 0.314 to 0.694 t/hm^2^ and nRMSE ranging from 7.28% to 17.23%. By coupling AIH and VIs features, the highest accuracy was achieved in estimating winter wheat AGB. The R^2^ increased to 0.728-0.925, indicating that the model fitting performance was good. Simultaneously, the RMSE decreased to 0.197-0.617 t/hm^2^ and nRMSE decreased to 4.58%-16.58%. This indicated that the addition of AIH features played a positive role in improving the accuracy of the AGB estimation model, reducing estimation errors.

**Table 3:** Estimation accuracy of wheat AGB with different features and their combinations.

Growth stage		Jointing stage			Heading stage			Grain filling stage		
Features		AIH	VIs	VIs+AIH	AIH	VIs	VIs+AIH	AIH	VIs	VIs+AIH
RFR	R^2^	0.60	0.89	0.93	0.51	0.84	0.90	0.55	0.80	0.90
	RMSE	0.59	0.32	0.20	0.78	0.45	0.35	0.86	0.60	0.37
	nRMSE(%)	25.5	13.53	10.64	15.67	8.88	6.96	10.57	7.28	4.58
XGBoost	R^2^	0.58	0.85	0.91	0.58	0.81	0.89	0.63	0.78	0.82
	RMSE	0.52	0.31	0.28	0.66	0.40	0.31	0.63	0.53	0.47
	nRMSE(%)	17.67	8.54	7.40	13.73	10.15	7.84	12.39	10.05	8.92
GBRT	R^2^	0.47	0.82	0.89	0.52	0.81	0.84	0.45	0.75	0.86
	RMSE	0.68	0.41	0.31	0.72	0.49	0.42	0.82	0.56	0.43
	nRMSE(%)	19.4	11.42	8.91	15.22	11.17	10.0	15.57	10.75	8.00
SVR	R^2^	0.46	0.87	0.88	0.46	0.82	0.86	0.42	0.71	0.74
	RMSE	0.69	0.35	0.33	0.83	0.44	0.4	0.89	0.64	0.60
	nRMSE(%)	29.54	14.65	13.29	16.67	8.81	7.86	10.78	7.74	7.43
RR	R^2^	0.42	0.81	0.83	0.50	0.75	0.77	0.44	0.69	0.73
	RMSE	0.72	0.41	0.39	0.74	0.52	0.49	0.76	0.69	0.62
	nRMSE(%)	31.23	17.23	16.58	14.66	10.42	9.81	9.11	8.45	7.48

**Figure 9:**
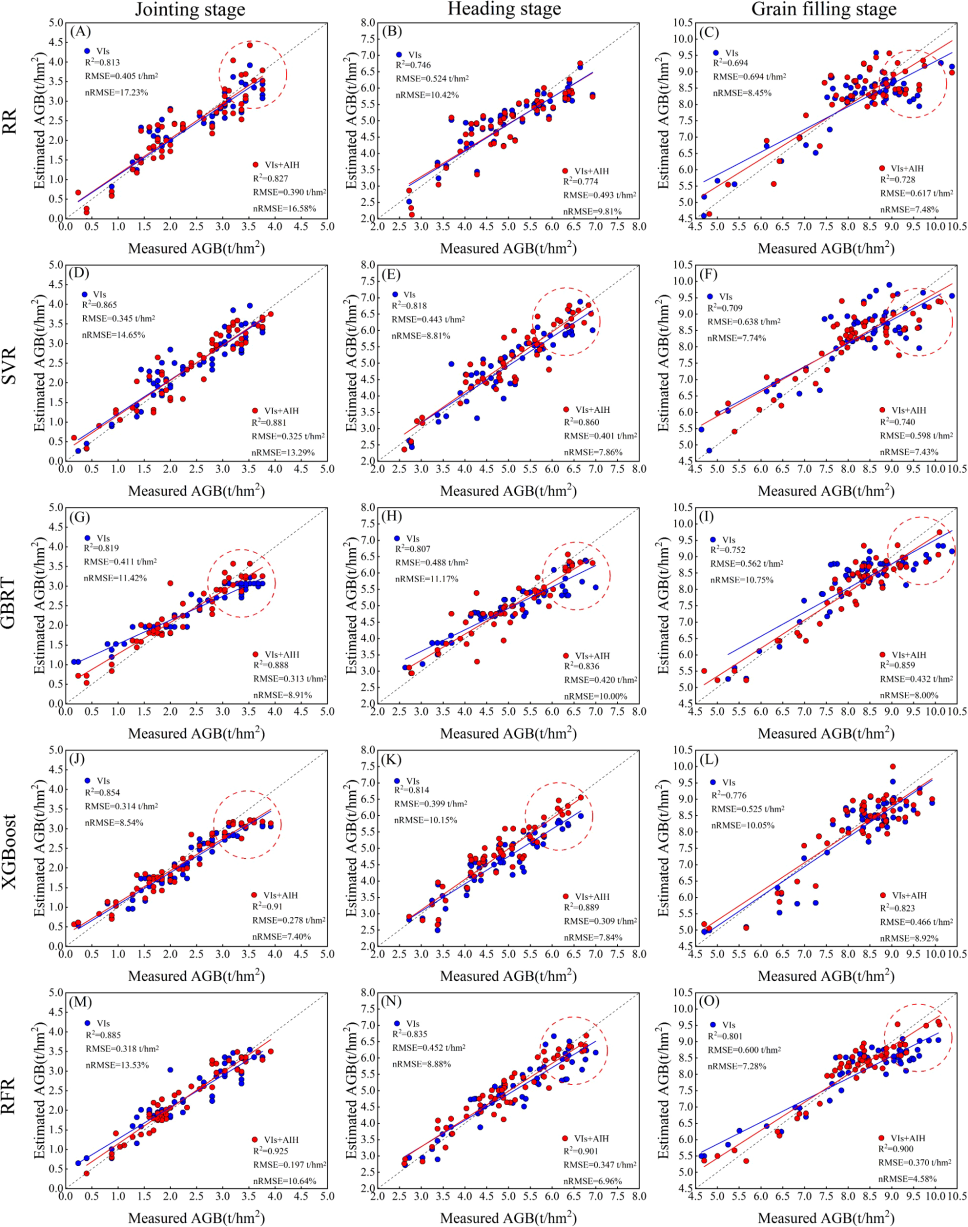
The scatter plots depicted the AGB estimates obtained through five machine learning algorithms (RFR, XGBoost, GBRT, SVR, and RR) at various growth stages. The corresponding columns represented the regression scatter plots for the jointing stage, heading stage and grain filling stage. **(A–C)** RR. **(D–F)** SVR. **(G–I)** GBRT. **(J–L)** XGBoost. **(M–O)** RFR. The dashed lines indicate the expected 1:1 relationship.

**Figure 9** displays the scatter plot distributions of regression predictions on different growth stages in the test set, considering both individual VIs features and feature combinations. The figure distinctly reflected the consistency between the predicted values of wheat AGB and the measured values. In comparison to the relatively scattered distribution of sample points in SVR and RR models, the sample points of RFR, XGBoost and GBRT models were concentrated around the 1:1 regression line, indicated its superior regression performance. The red circles in the figure indicated that at different growth stages, as the measured AGB values increased, the predicted values exhibited slow growth and lower than the measured values, suggesting the occurrence of spectral saturation. In AGB estimation based on the fusion of multiple features, it was discovered that the model accuracy improved, mitigating the spectral saturation phenomenon to some extent.

### 3.3 Performance evaluation of different machine learning algorithms

**Figure 10:**
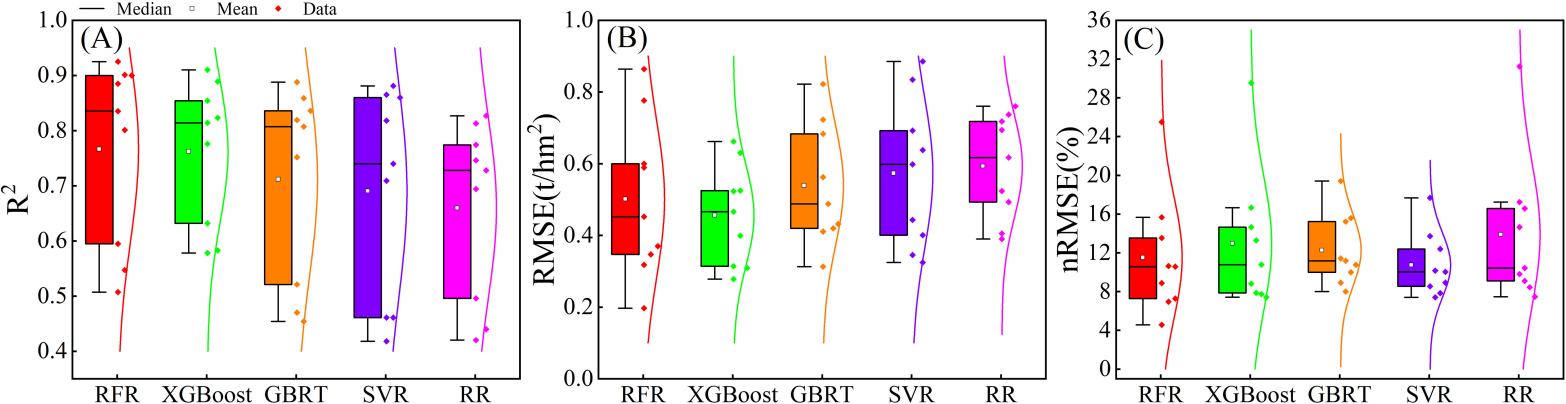
Accuracy of AGB estimation using different machine learning algorithms. **(A)** R^2^. **(B)** RMSE. **(C)** nRMSE.

For evaluating the performance and generalization effects of different machine learning algorithms, a comparative analysis was conducted on the accuracy of five algorithms: RFR, XGBoost, GBRT, SVR and RR (**Figure 10**). The results clearly demonstrated that machine learning algorithms based on decision tree methods exhibited higher accuracy and smaller errors compared to the other two algorithms. This reflected the significant advantage of decision tree based regression algorithms in handling non-linear relationships without the need for feature scaling. The median values of R^2^ were consistently higher than 0.80, RMSE median values were consistently lower than 0.49t/hm^2^, and nRMSE median values were consistently lower than 12%. Among machine learning algorithms, the RFR algorithm was considered the best, slightly outperforming the XGBoost algorithm. The R^2^ median for the RFR algorithm was 0.835 and the RMSE median was 0.452 t/hm^2^. The least performing algorithm was RR, a traditional machine learning algorithm, with R^2^ median was 0.728 and RMSE median was 0.617 t/hm^2^. Overall, RMSE and nRMSE exhibited relative stability across the five machine learning algorithms, indicating that the differences between the original and estimated values of biomass during various growth stages show no significant variations.

## 4 Discussion

### 4.1 Extraction of winter wheat crop height

As a critical growth parameter for crops, the extraction of crop height, especially for winter wheat, has been the focus of numerous studies (Jia et al., 2022; Ma et al., 2022). In comparison to orthophoto map, oblique photography technology has revolutionized the limitations of capturing images only from a vertical perspective. Based on the planning of five flight paths, oblique photography technology tilts the camera at a specific angle to comprehensively capture target images. With its features of extensive coverage, high accuracy, and high resolution, it provides an intuitive representation of crop texture, location, height and other information, making it highly favored among surveying and mapping professionals. However, the widespread adoption of LiDAR has faced constraints due to its expensive cost and limitations, such as the inability to capture color information of objects (Zhang et al., 2021). Grüner et al. successfully estimated crop height in grassland vegetation using a UAV equipped with an RGB camera (Grüner et al., 2019). In previous studies (Kawamura et al., 2020; Bhandari et al., 2023), the use of CHM for extracting crop height has been quite common. However, there is limited research on the comparative analysis of the accuracy of different crop height extraction methods. The mean method for extracting crop height may result in less accurate crop height extraction due to issues such as image matching, the sparse nature of wheat, the high resolution of the crop DSM images, and the influence of bare soil. This is consistent with the findings of Chang et al (Chang et al., 2017). The AIH method involves normalizing the point cloud of vegetation points with absolute elevations. The AIH values are then employed as the crop height for wheat plants, mitigating the impact of variations in wheat density. Moreover, different AIH exhibit varied performance in crop height extraction. In a study based on four different AIH values—80%, 90%, 95% and 99%—it was observed that as the AIH increases, the accuracy of crop height extraction shows a trend of initially increasing and then decreasing. The highest accuracy is achieved at the 95% AIH (Hütt et al., 2023). This differs from the findings of Lu et al (Lu et al., 2021), possibly attributed to their study focus on summer maize crop height extraction. The differences in the shape of the canopy top, where the canopy top of summer maize is more spike-like, could be a contributing factor.

### 4.2 Contribution of different features in AGB estimation

In previous studies on winter wheat AGB estimation, the utilization of VIs for AGB estimation has become widespread. However, different features may have varying impacts on AGB estimation, and relying solely on VIs extracted from RGB images may result in suboptimal accuracy (Lu et al., 2019; Wang et al., 2022). Su et al. (Su et al., 2024a) have demonstrated that spectral features in the near-infrared band can accurately capture the spectral differences of SPAD during the growth period of winter wheat in SPAD estimation based on UAV multispectral imagery. To enhance estimation accuracy, this study employed VIs extracted from RGB and multispectral imagery as features. The study indicates that during various growth stages, VIs associated with the NIR band exhibit higher feature importance in AGB estimation. Which reflects that the NIR band enhances the contrast of vegetation vitality, aligning with prior research findings (Tilly et al., 2014; Wang et al., 2023). In the random forest variable importance analysis of vegetation index features, it was found that the importance of RTVI-CORE, RDVI-REG, LCI and OSAVI all exceeded 8.5%. These sensitive vegetation indices are closely associated with the NIR band, further confirming the significance of the NIR band in monitoring wheat growth. The NIR wavelength region is strongly correlated with the internal structure and biochemical composition of crop leaves. Crop cell structures reflect a high proportion of NIR spectra, making this wavelength region highly sensitive to crop health conditions (Su et al., 2024b). With the progression of the growth stages, the predicted values are slightly lower than the actual values, indicating the occurrence of spectral saturation. Neumann et al. contend that the occurrence of spectral saturation limits the accuracy of AGB estimation in densely vegetated areas (Neumann et al., 2020). During the jointing and early grain filling stage, wheat exhibits robust growth with high leaf overlap, leading to the manifestation of vegetation spectral saturation. Furthermore, in the later stages of wheat growth, AGB is composed of leaves, stems, and spikes. The spectral information of the canopy is primarily influenced by both leaves and spikes. Relying solely on spectral information obtained from the canopy for AGB estimation may introduce some bias (Lu et al., 2019).

Crop height provides an intuitive representation of the vertical structure distribution of crop plants. Previous studies have demonstrated that crop height for wheat (Lu et al., 2019; Walter et al., 2019) and maize (Shu et al., 2023) exhibit correlation with AGB. Over 90% AIH reflects the point cloud distribution of the vegetation canopy, offering a relatively accurate representation of crop height. AIH and AGB exhibit a moderate correlation (Walter et al., 2019). In the feature importance analysis, the majority of AIH features exhibit high importance in the AGB estimation models, with only 40% AIH features having lower importance. This phenomenon may be attributed to the fact that 40% AIH is located roughly in the middle of the vertical distribution of the crop. Due to the higher density of canopy leaves, there is a lower point cloud count in that position, making it less accurate in representing crop height. By coupling VIs and AIH features, the limitations of spectral saturation can be mitigated. Zhu et al. (2023b) developed a three-dimensional conceptual model (3DCM) based on plant height and vegetation coverage to mitigate the spectral saturation effect in wheat AGB estimation. They found that the 3DCM model outperformed traditional vegetation index models and conventional multi-feature combination models, with the highest accuracy at the wheat nodulation stage, verifying the feasibility of synergistic use of UAV height information with VIs. In practical applications, integrating features extracted from multiple remote sensing data sources can better address the characteristics of different regions and vegetation types, enhancing the applicability and accuracy of AGB estimation. The addition of AIH features had a positive impact on all five models during various growth stages, mitigating spectral saturation. This indicates that crop height is a crucial indicator of wheat growth and development. The dynamic changes in AIH provide an intuitive reflection of the growth status of wheat (Niu et al., 2019). Furthermore, multivariate features incorporating spectral and spatiotemporal information of the wheat population exhibit superior predictive capabilities for AGB compared to single-variable features (Liu et al., 2018). By delving into the multi-dimensional information of vegetation, a better understanding of the growth patterns of vegetation can be achieved, providing robust support for agricultural production and ecological environment monitoring.

### 4.3 Comparison of AGB modeling methods

This study evaluated the performance of five machine learning regression algorithms—RFR, XGBoost, GBRT, SVR and RR in estimating AGB, all of which achieved satisfactory accuracy. Non-linear regression models, specifically RFR, XGBoost and GBRT, exhibited superior accuracy compared to linear regression models SVR and RR. This discrepancy was attributed to the intricate non-linear relationship between remote sensing data and biomass. Relying solely on linear regression models was highly susceptible to the influence of outliers, rendering it incapable of producing accurate predictive results (Lu et al., 2019; Tatsumi et al., 2021). Among the five machine learning algorithms, the RFR model outperformed others, displaying the highest coefficient of determination and the lowest errors. This could be credited to the model’s aggregation of multiple decision trees, effectively mitigating the impact of noise and outliers. However, the RFR model has some drawbacks, such as high computational demands and a lack of interpretability. Moreover, introducing extra randomness in sample extraction and feature selection during model construction reduced the risk of overfitting, enhancing model stability and estimation capabilities. RFR, XGBoost and GBRT, all constructed based on multiple decision trees, achieved high accuracy in estimating wheat AGB. XGBoost and GBRT showed slightly lower estimation performance compared to RFR (Wang et al., 2022). Regression algorithms based on decision trees can achieve higher estimation accuracy due to its continuous iteration to reduce errors, autonomous feature selection capabilities for handling high-dimensional data, strong generalization ability and robustness (Liu et al., 2022; Poudyal et al., 2022). Due to the interdependence among weak learners, GBRT is challenging to train in parallel and may not perform as well as neural networks when handling high-dimensional data. Therefore, employing some local parallelization techniques within decision trees during training can improve the model’s training speed. As an efficient implementation of GBRT, XGBoost introduces feature subsampling, which reduces overfitting and computational load. However, during node splitting, it still needs to traverse the dataset, and storing the feature values and their corresponding sample gradient statistics requires twice the memory. However, SVR is not well-suited for handling large-scale datasets, is sensitive to parameter tuning, has high computational demands, and has lower accuracy in the presence of high sample noise. In contrast, RR exhibited the lowest estimation accuracy in this study. RR was highly sensitive to noise and outliers in the data, and the decrease in estimation accuracy could be attributed to the presence of data noise and nonlinear relationships. Therefore, constructing accurate and compact decision tree machine learning algorithms lays a solid foundation for agricultural production and growth monitoring (Ji et al., 2023). This method provided a quantitative means to evaluate the AGB of wheat with a simple and efficient operation and had the potential to be used on a large scale.

### 4.4 Limits and significance of the study

UAVs with their advantages of maneuverability, convenience and speed, were widespread applications in agricultural crop information monitoring, providing real-time and accurate decision support for agricultural production (Ji et al., 2019). The crop height serves as a crucial indicator of crop growth status, and the real-time and accurate prediction of crop height is essential for monitoring the overall crop development. The utilization of AIH extracted from point cloud data has achieved considerable accuracy in crop height estimation. However, RGB point clouds obtained through oblique photogrammetry mainly capture the top of the vegetation canopy, with limited penetration ability and susceptibility to significant environmental influences (Li et al., 2016). In future research, the approach of low-altitude cross-circular hovering oblique photography can be adopted to construct a three-dimensional point cloud, achieving precise acquisition of three-dimensional point cloud data (Hütt et al., 2023). When estimating AGB using VIs extracted from RGB and multispectral imagery, spectral saturation occurred during the grain filling period. However, by incorporating multiple AIH features into the model, the issue of spectral saturation was alleviated (Zhang et al., 2023). Therefore, coupling feature extraction from multiple remote sensing data sources can enhance AGB estimation accuracy. In future research, exploring the impact of other features on estimation accuracy could be considered.

A gradual increase in AGB was observed as the crop progressed through growth and development. The complete growth cycle of winter wheat spans from seed germination to the production of new mature seeds, encompassing multiple growth stages. However, this experiment focused solely on crop height and AGB estimation during the jointing, heading, and grain filling stages of winter wheat, potentially resulting in AGB changes is localized. Future research should systematically investigate crop height and AGB variations across all growth stages of the wheat lifecycle to uncover the rhythms in wheat growth (Zhai et al., 2023). Previous studies have indicated that estimating corn AGB by multiplying Leaf Area Index (LAI) with crop height performs well (Shu et al., 2023). Due to the lush foliage and higher plant density in the later stages of winter wheat growth, LAI shows little variation and was not studied in this experiment. Moreover, this experiment was conducted in a small-scale farmland in the north of Henan, which may limit the model’s applicability. Variations in factors such as temperature, soil type, entropy, precipitation, and daylight length in different regions, there are limitations in extending the wheat crop height and AGB estimation model to other areas. In future research, a comprehensive consideration of ground conditions and meteorological data, collection of more extensive data, and conducting cross-validation experiments on data from different regions should be performed to enhance the model’s generalization ability (Sun et al., 2020).

## 5 Conclusions

This study aims to evaluate and analyze the effectiveness of the mean method and AIH method in extracting crop height for winter wheat. Additionally, the accuracy of winter wheat AGB estimation was assessed using AIH, VIs and their combinations. Ultimately, the estimation performance of the five machine learning algorithms was compared and analyzed. The following conclusions were drawn:

(1) Crop height extraction methods based on UAV remote sensing data reveal that the crop height extraction method based on AIH is more accurate than the mean method across various growth stages. When comparing the performance of different AIH values in crop height extraction, it was discovered that the 95% AIH accurately represents crop height.
(2) In comparison to NDVI, kNDVI exhibited more higher feature importance. VIs correlated with the NIR band were more sensitive in monitoring crop growth conditions and demonstrated higher feature importance. Coupling VIs and AIH features for AGB estimation achieved higher accuracy compared to estimating AGB with a single feature. Additionally, embedding AIH features into the estimation model mitigated spectral saturation to some extent.
(3) Various machine learning algorithms showed different performance in estimating wheat AGB. Ensemble learning algorithms based on decision trees, represented by RFR, XGBoost and GBRT, consistently demonstrated higher accuracy compared to other linear machine learning algorithms. Among the five machine learning algorithms, RFR achieved the best estimation results at different growth stages.

In summary, the use of point cloud data obtained through the oblique photography technique provides an intuitive representation of crop height information. The coupling of multiple features and robust machine learning algorithms offer a new reference for estimating AGB in wheat. Leveraging multi-source remote sensing technology with UAVs meets the demand for convenient and efficient acquisition of crop growth information. This provides technical support for precision agriculture and decision-making in agricultural fields.

## Data Availability

The data analyzed in this study is subject to the following licenses/restrictions: The dataset is restricted to research for this paper only. Requests to access these datasets should be directed to YL, 212204020029@home.hpu.edu.cn.

## References

[B1] Abdollahpour S. Kosari-Moghaddam A. Bannayan M. (2020). Prediction of wheat moisture content at harvest time through ANN and SVR modeling techniques. Inf. Process. Agric. 7, 500–510. doi: 10.1016/j.inpa.2020.01.003

[B2] Bendig J. Yu K. Aasen H. Bolten A. Bennertz S. Broscheit J.. (2015). Combining UAV-based plant height from crop surface models, visible, and near infrared vegetation indices for biomass monitoring in barley. Int. J. Appl. Earth Observ. Geoinform. 39, 79–87. doi: 10.1016/j.jag.2015.02.012

[B3] Bhandari M. Chang A. Jung J. Ibrahim A. M. H. Rudd J. C. Baker S.. (2023). Unmanned aerial system-based high-throughput phenotyping for plant breeding. Plant Phenome J. 6, e20058. doi: 10.1002/ppj2.20058

[B4] Blanco V. Blaya-Ros P. J. Castillo C. Soto-Vallés F. Torres-Sánchez R. Domingo R. (2020). Potential of UAS-based remote sensing for estimating tree water status and yield in sweet cherry trees. Remote Sens. 12, 2359. doi: 10.3390/rs12152359

[B5] Camps-Valls G. Campos-Taberner M. Moreno-Martínez Á. Walther S. Duveiller G. Cescatti A.. (2021). A unified vegetation index for quantifying the terrestrial biosphere. Sci. Adv. 7, eabc7447. doi: 10.1126/sciadv.abc7447 33637524 PMC7909876

[B6] Chang A. Jung J. Maeda M. M. Landivar J. (2017). Crop height monitoring with digital imagery from Unmanned Aerial System (UAS). Comput. Electron. Agric. 141, 232–237. doi: 10.1016/j.compag.2017.07.008

[B7] Chen Q. (2015). Modeling aboveground tree woody biomass using national-scale allometric methods and airborne lidar. ISPRS J. Photogram. Remote Sens. 106, 95–106. doi: 10.1016/j.isprsjprs.2015.05.007

[B8] Chen P. (2020). Estimation of winter wheat grain protein content based on multisource data assimilation. Remote Sens. 12, 3201. doi: 10.3390/rs12193201

[B9] Ehlers D. Wang C. Coulston J. Zhang Y. Pavelsky T. Frankenberg E.. (2022). Mapping forest aboveground biomass using multisource remotely sensed data. Remote Sens. 14, 1115. doi: 10.3390/rs14051115

[B10] Feng D. Xu W. He Z. Zhao W. Yang M. (2020). Advances in plant nutrition diagnosis based on remote sensing and computer application. Neural Comput. Applic 32, 16833–16842. doi: 10.1007/s00521-018-3932-0

[B11] Fu Y. Yang G. Wang J. Song X. Feng H. (2014). Winter wheat biomass estimation based on spectral indices, band depth analysis and partial least squares regression using hyperspectral measurements. Comput. Electron. Agric. 100, 51–59. doi: 10.1016/j.compag.2013.10.010 23905343

[B12] Gitelson A. A. Gritz Y. Merzlyak M. N. (2003). Relationships between leaf chlorophyll content and spectral reflectance and algorithms for non-destructive chlorophyll assessment in higher plant leaves. J. Plant Physiol. 160, 271–282. doi: 10.1078/0176-1617-00887 12749084

[B13] Grüner E. Astor T. Wachendorf M. (2019). Biomass prediction of heterogeneous temperate grasslands using an sfM approach based on UAV imaging. Agronomy 9, 54. doi: 10.3390/agronomy9020054

[B14] Hague T. Tillett N. D. Wheeler H. (2006). Automated crop and weed monitoring in widely spaced cereals. Precis. Agric. 7, 21–32. doi: 10.1007/s11119-005-6787-1

[B15] Huang X. Lin D. Mao X. Zhao Y. (2023). Multi-source data fusion for estimating maize leaf area index over the whole growing season under different mulching and irrigation conditions. Field Crops Res. 303, 109111. doi: 10.1016/j.fcr.2023.109111

[B16] Huang J. Sedano F. Huang Y. Ma H. Li X. Liang S.. (2016). Assimilating a synthetic Kalman filter leaf area index series into the WOFOST model to improve regional winter wheat yield estimation. Agric. For. Meteorol. 216, 188–202. doi: 10.1016/j.agrformet.2015.10.013

[B17] Hütt C. Bolten A. Hüging H. Bareth G. (2023). UAV liDAR metrics for monitoring crop height, biomass and nitrogen uptake: A case study on a winter wheat field trial. PFG 91, 65–76. doi: 10.1007/s41064-022-00228-6

[B18] Jayathunga S. Owari T. Tsuyuki S. (2018). Evaluating the performance of photogrammetric products using fixed-wing UAV imagery over a mixed conifer–broadleaf forest: comparison with airborne laser scanning. Remote Sens. 10, 187. doi: 10.3390/rs10020187

[B19] Ji Y. Liu R. Xiao Y. Cui Y. Chen Z. Zong X.. (2023). Faba bean above-ground biomass and bean yield estimation based on consumer-grade unmanned aerial vehicle RGB images and ensemble learning. Precis. Agric. 24, 1439–1460. doi: 10.1007/s11119-023-09997-5

[B20] Ji J. Zhao Y. Zhou X. Xuan K. Wang W. Liu J.. (2019). Adcancement in application of UAV remote sensing to monitoring of farmlands. Acta Pedol. Sin. 56, 773–784. doi: 10.6046/gtzyyg.2019.01.20

[B21] Jia A. Dong T. Zhang Y. Zhu B. Sun Y. Wu Y.. (2022). Recognition of field-grown tobacco plant type characteristics based on three-dimensional point cloud and ensemble learning. J. Zhejiang Univ. (Agriculture Life Sciences) 48, 393–402. doi: 10.3785/j.issn.1008-9209.2021.05.173

[B22] Jiang Z. Huete A. Didan K. Miura T. (2008). Development of a two-band enhanced vegetation index without a blue band. Remote Sens. Environ. 112, 3833–3845. doi: 10.1016/j.rse.2008.06.006

[B23] Jimenez-Berni J. A. Deery D. M. Rozas-Larraondo P. Condon A. Rebetzke G. J. James R. A.. (2018). High throughput determination of plant height, ground cover, and above-ground biomass in wheat with liDAR. Front. Plant Sci. 9. doi: 10.3389/fpls.2018.00237 PMC5835033 29535749

[B24] Kawamura K. Asai H. Yasuda T. Khanthavong P. Soisouvanh P. Phongchanmixay S. (2020). Field phenotyping of plant height in an upland rice field in Laos using low-cost small unmanned aerial vehicles (UAVs). Plant Product. Sci. 23, 452–465. doi: 10.1080/1343943X.2020.1766362

[B25] Kotivuori E. Kukkonen M. Mehtätalo L. Maltamo M. Korhonen L. Packalen P. (2020). Forest inventories for small areas using drone imagery without *in-situ* field measurements. Remote Sens. Environ. 237, 111404. doi: 10.1016/j.rse.2019.111404

[B26] Kross A. McNairn H. Lapen D. Sunohara M. Champagne C. (2015). Assessment of RapidEye vegetation indices for estimation of leaf area index and biomass in corn and soybean crops. Int. J. Appl. Earth Observ. Geoinform. 34, 235–248. doi: 10.1016/j.jag.2014.08.002

[B27] Lee K.-J. Lee B.-W. (2013). Estimation of rice growth and nitrogen nutrition status using color digital camera image analysis. Eur. J. Agron. 48, 57–65. doi: 10.1016/j.eja.2013.02.011

[B28] Li W. Huang J. Qi Y. Liu X. Liu J. Mao Z.. (2023). Meta-analysis of soil microbial biomass carbon content and its influencing factors under soil erosion. Ecol. Environ. 32, 47–55. doi: 10.16258/j.cnki.1674-5906.2023.01.006

[B29] Li W. Niu Z. Chen H. Li D. Wu M. Zhao W. (2016). Remote estimation of canopy height and aboveground biomass of maize using high-resolution stereo images from a low-cost unmanned aerial vehicle system. Ecol. Indic. 67, 637–648. doi: 10.1016/j.ecolind.2016.03.036

[B30] Li C. Shi J. Ma C. Cui Y. Wang Y. Li Y. (2021). Estimation of chlorophyll content in winter wheat based on wavelet transform and fractional differential. Trans. Chin. Soc. Agric. Machinery 52, 172–182. doi: 10.6041/j.issn.1000-1298.2021.03.019

[B31] Li J. Wang M. Wang Y. Zhan S. Duan P. (2022). Vegetation information classification method considering UAV image point cloud characteristics. Ecol. Sci. 41, 11–18. doi: 10.14108/j.cnki.1008-8873.2022.05.002

[B32] Li M. Wu B. Yan C. Zhou W. (2004). Estimation of vegetation fraction in the upper basin of miyun reservoir by remote sensing. Resour. Sci. 26(4), 153–159. doi: 10.1007/BF02973453

[B33] Li Y. Yu H. Wang Y. Wu J. Yang L. (2019). Classification of urban vegetation based on unmanned aerial vehicle reconstruction point cloud and image. Remote Sens. Land Resour. 31, 149–155. doi: 10.6046/gtzyyg.2019.01.20

[B34] Liu Y. Wang S. Wang X. Chen B. Chen J. Wang J.. (2022). Exploring the superiority of solar-induced chlorophyll fluorescence data in predicting wheat yield using machine learning and deep learning methods. Comput. Electron. Agric. 192, 106612. doi: 10.1016/j.compag.2021.106612

[B35] Liu C. Yang G. Li Z. Tang F. Wang J. Zhang C.. (2018). Biomass estimation in winter wheat by UAV spectral information and texture information fusion. Scientia Agricult. Sin. 51, 3060–3073. doi: 10.6046/gtzyyg.2019.01.20

[B36] Liu D. Yang F. Liu S. (2021). Estimating wheat fractional vegetation cover using a density peak k-means algorithm based on hyperspectral image data. J. Integr. Agric. 20, 2880–2891. doi: 10.1016/S2095-3119(20)63556-0

[B37] Lu J. Cheng D. Geng C. Zhang Z. Xiang Y. Hu T. (2021). Combining plant height, canopy coverage and vegetation index from UAV-based RGB images to estimate leaf nitrogen concentration of summer maize. Biosyst. Eng. 202, 42–54. doi: 10.1016/j.biosystemseng.2020.11.010

[B38] Lu N. Zhou J. Han Z. Li D. Cao Q. Yao X.. (2019). Improved estimation of aboveground biomass in wheat from RGB imagery and point cloud data acquired with a low-cost unmanned aerial vehicle system. Plant Methods 15, 17. doi: 10.1186/s13007-019-0402-3 30828356 PMC6381699

[B39] Ma X. Wei B. Guan H. Yu S. (2022). A method of calculating phenotypic traits for soybean canopies based on three-dimensional point cloud. Ecol. Inf. 68, 101524. doi: 10.1016/j.ecoinf.2021.101524

[B40] Madec S. Baret F. De Solan B. Thomas S. Dutartre D. Jezequel S.. (2017). High-throughput phenotyping of plant height: comparing unmanned aerial vehicles and ground liDAR estimates. Front. Plant Sci. 8. doi: 10.3389/fpls.2017.02002 PMC5711830 29230229

[B41] Maimaitijiang M. Sagan V. Sidike P. Maimaitiyiming M. Hartling S. Peterson K. T.. (2019). Vegetation Index Weighted Canopy Volume Model (CVMVI) for soybean biomass estimation from Unmanned Aerial System-based RGB imagery. ISPRS J. Photogram. Remote Sens. 151, 27–41. doi: 10.1016/j.isprsjprs.2019.03.003

[B42] Maimaitiyiming M. Sagan V. Sidike P. Kwasniewski M. (2019). Dual activation function-based extreme learning machine (ELM) for estimating grapevine berry yield and quality. Remote Sens. 11, 740. doi: 10.3390/rs11070740

[B43] Malambo L. Popescu S. C. Murray S. C. Putman E. Pugh N. A. Horne D. W.. (2018). Multitemporal field-based plant height estimation using 3D point clouds generated from small unmanned aerial systems high-resolution imagery. Int. J. Appl. Earth Observ. Geoinform. 64, 31–42. doi: 10.1016/j.jag.2017.08.014

[B44] Meng S. Pang Y. Zhang Z. Jia W. Li Z. (2016). Mapping aboveground biomass using texture indices from aerial photos in a temperate forest of northeastern China. Remote Sens. 8, 230. doi: 10.3390/rs8030230

[B45] Meyer G. E. Neto J. C. (2008). Verification of color vegetation indices for automated crop imaging applications. Comput. Electron. Agric. 63, 282–293. doi: 10.1016/j.compag.2008.03.009

[B46] Neumann E. K. Djambazova K. V. Caprioli R. M. Spraggins J. M. (2020). Multimodal imaging mass spectrometry: next generation molecular mapping in biology and medicine. J. Am. Soc Mass Spectrom. 31, 2401–2415. doi: 10.1021/jasms.0c00232 32886506 PMC9278956

[B47] Niu Y. Zhang L. Zhang H. Han W. Peng X. (2019). Estimating above-ground biomass of maize using features derived from UAV-based RGB imagery. Remote Sens. 11, 1261. doi: 10.3390/rs11111261

[B48] Pinto J. Powell S. Peterson R. Rosalen D. Fernandes O. (2020). Detection of defoliation injury in peanut with hyperspectral proximal remote sensing. Remote Sens. 12, 3828. doi: 10.3390/rs12223828

[B49] Poudyal C. Costa L. F. Sandhu H. Ampatzidis Y. Odero D. C. Arbelo O. C.. (2022). Sugarcane yield prediction and genotype selection using unmanned aerial vehicle-based hyperspectral imaging and machine learning. Agron. J. 114, 2320–2333. doi: 10.1002/agj2.21133

[B50] Roujean J.-L. Breon F.-M. (1995). Estimating PAR absorbed by vegetation from bidirectional reflectance measurements. Remote Sens. Environ. 51, 375–384. doi: 10.1016/0034-4257(94)00114-3

[B51] Sagan V. Maimaitijiang M. Sidike P. Eblimit K. Peterson K. Hartling S.. (2019). UAV-based high resolution thermal imaging for vegetation monitoring, and plant phenotyping using ICI 8640 P, FLIR vue pro R 640, and thermoMap cameras. Remote Sens. 11, 330. doi: 10.3390/rs11030330

[B52] Shah S. H. Angel Y. Houborg R. Ali S. McCabe M. F. (2019). A random forest machine learning approach for the retrieval of leaf chlorophyll content in wheat. Remote Sens. 11, 920. doi: 10.3390/rs11080920

[B53] Shu M. Li Q. Ghafoor A. Zhu J. Li B. Ma Y. (2023). Using the plant height and canopy coverage to estimation maize aboveground biomass with UAV digital images. Eur. J. Agron. 151, 126957. doi: 10.1016/j.eja.2023.126957

[B54] Singh B. Kumar S. Elangovan A. Vasht D. Arya S. Duc N. T.. (2023). Phenomics based prediction of plant biomass and leaf area in wheat using machine learning approaches. Front. Plant Sci. 14. doi: 10.3389/fpls.2023.1214801 PMC10337996 37448870

[B55] Su X. Nian Y. Shaghaleh H. Hamad A. Yue H. Zhu Y.. (2024a). Combining features selection strategy and features fusion strategy for SPAD estimation of winter wheat based on UAV multispectral imagery. Front. IN Plant Sci. 15. doi: 10.3389/fpls.2024.1404238 PMC11116665 38799101

[B56] Su X. Nian Y. Yue H. Zhu Y. Li J. Wang W.. (2024b). Improving wheat leaf nitrogen concentration (LNC) estimation across multiple growth stages using feature combination indices (FCIs) from UAV multispectral imagery. AGRONOMY-BASEL 14, 1052. doi: 10.3390/agronomy14051052

[B57] Sun Q. Guan L. Jiao Q. Liu X. Dai H. (2021). Research on retrieving biomass of winter wheat based on fusing vegetation index. Remote Sens. Technol. Appl. 36, 391–399. doi: 10.11873/j.issn.1004-0323.2021.2.0391

[B58] Sun L. Qian Y. Wu S. Deng H. Shen Y. Tao S.. (2020). Dynamic monitoring of winter wheat growth in 2020 in China. Anhui Agric.Sci 48, 230–233 + 273. doi: 10.6046/gtzyyg.2019.01.20

[B59] Tatsumi K. Igarashi N. Mengxue X. (2021). Prediction of plant-level tomato biomass and yield using machine learning with unmanned aerial vehicle imagery. Plant Methods 17, 77. doi: 10.1186/s13007-021-00761-2 34266447 PMC8281694

[B60] Tilly N. Aasen H. Bareth G. (2015). Fusion of plant height and vegetation indices for the estimation of barley biomass. Remote Sens. 7, 11449–11480. doi: 10.3390/rs70911449

[B61] Tilly N. Hoffmeister D. Cao Q. Huang S. Lenz-Wiedemann V. Miao Y.. (2014). Multitemporal crop surface models: accurate plant height measurement and biomass estimation with terrestrial laser scanning in paddy rice. J. Appl. Remote Sens 8, 83671. doi: 10.1117/1.JRS.8.083671

[B62] Wallace L. Hillman S. Reinke K. Hally B. (2017). Non-destructive estimation of above-ground surface and near-surface biomass using 3D terrestrial remote sensing techniques. Methods Ecol. Evol. 8, 1607–1616. doi: 10.1111/2041-210X.12759

[B63] Walsh O. S. Shafian S. Marshall J. M. Jackson C. Walsh W. L. (2018). Assessment of UAV based vegetation indices for nitrogen concentration estimation in spring wheat. Adv. Remote Sens. 07, 71–90. doi: 10.4236/ars.2018.72006

[B64] Walter J. D. C. Edwards J. McDonald G. Kuchel H. (2019). Estimating biomass and canopy height with liDAR for field crop breeding. Front. Plant Sci. 10. doi: 10.3389/fpls.2019.01145 PMC6775483 31611889

[B65] Wang G. Liu S. Liu T. Fu Z. Yu J. Xue B. (2019). Modelling above-ground biomass based on vegetation indexes: a modified approach for biomass estimation in semi-arid grasslands. Int. J. Remote Sens. 40, 3835–3854. doi: 10.1080/01431161.2018.1553319

[B66] Wang H. Xiang Y. Li W. Shi H. Wang X. Zhao X. (2023). Estimation of winter rapeseed above-ground biomass based on UAV multi-spectral remote sensing. Trans. Chin. Soc. Agric. Machinery 54, 218–229. doi: 10.1007/BF02973453

[B67] Wang F. Yang M. Ma L. Zhang T. Qin W. Li W.. (2022). Estimation of above-ground biomass of winter wheat based on consumer-grade multi-spectral UAV. Remote Sens. 14, 1251. doi: 10.3390/rs14051251

[B68] Wei Q. Wang F. Zhang Q. Chen D. Lu X. (2021). Dynamic monitoring method of winter wheat biomass based on near earth remote sensing. Shandong Agric. Sci. 53, 132–138. doi: 10.14083/j.issn.1001-4942.2021.03.023

[B69] Wen Y. Lü J. Ma Q. Zhang P. Xu R. (2022). Study on inversion of forest biomass by LiDAR and hyperspectral. Bull. Survey. Mapp. 0(7), 38–42. doi: 10.13474/j.cnki.11-2246.2022.0200

[B70] Xiao Y. Zhao W. Zhou D. Gong H. (2014). Sensitivity analysis of vegetation reflectance to biochemical and biophysical variables at leaf, canopy, and regional scales. IEEE Trans. Geosci. Remote Sens. 52, 4014–4024. doi: 10.1109/TGRS.2013.2278838

[B71] Xu L. Zhou L. Meng R. Zhao F. Lv Z. Xu B.. (2022). An improved approach to estimate ratoon rice aboveground biomass by integrating UAV-based spectral, textural and structural features. Precis. Agric. 23, 1276–1301. doi: 10.1007/s11119-022-09884-5

[B72] Yang J. Ma X. Fan Y. Liu Z. Li H. Sun Y. (2020). Spring wheat field management techniques in xinxiang city. Modern Agric. Sci. Technol. 2020(20), 46–47. doi: 10.3969/j.issn.1007-5739.2020.20.018

[B73] Yue J. Yang G. Tian Q. Feng H. Xu K. Zhou C. (2019). Estimate of winter-wheat above-ground biomass based on UAV ultrahigh-ground-resolution image textures and vegetation indices. ISPRS J. Photogram. Remote Sens. 150, 226–244. doi: 10.1016/j.isprsjprs.2019.02.022

[B74] Zhai W. Li C. Cheng Q. Mao B. Li Z. Li Y.. (2023). Enhancing wheat above-ground biomass estimation using UAV RGB images and machine learning: multi-feature combinations, flight height, and algorithm implications. Remote Sens. 15, 3653. doi: 10.3390/rs15143653

[B75] Zhang C. Denka S. Cooper H. Mishra D. R. (2018). Quantification of sawgrass marsh aboveground biomass in the coastal Everglades using object-based ensemble analysis and Landsat data. Remote Sens. Environ. 204, 366–379. doi: 10.1016/j.rse.2017.10.018

[B76] Zhang M. Liu T. Sun C. (2023). Wheat biomass estimation based on UAV hyperspectral data. Anhui Agric.Sci 51, 182–186 + 189. doi: 10.3969/j.issn.0517-6611.2023.17.041

[B77] Zhang Y.-W. Wang T. Guo Y. Skidmore A. Zhang Z. Tang R.. (2022). Estimating community-level plant functional traits in a species-rich alpine meadow using UAV image spectroscopy. Remote Sens. 14, 3399. doi: 10.3390/rs14143399

[B78] Zhang J. Xie T. Yang W. Zhou G. (2021). Research status and prospect on height estimation of field crop using near-field remote sensing technology. Smart Agric. 3, 1–15. doi: 10.12133/j.smartag.2021.3.1.202102-SA033

[B79] Zhang X. Zhang F. Qi Y. Deng L. Wang X. Yang S. (2019). New research methods for vegetation information extraction based on visible light remote sensing images from an unmanned aerial vehicle (UAV). Int. J. Appl. Earth Observ. Geoinform. 78, 215–226. doi: 10.1016/j.jag.2019.01.001

[B80] Zhu Y. Liu J. Tao X. Su X. Li W. Zha H.. (2023b). A three-dimensional conceptual model for estimating the above-ground biomass of winter wheat using digital and multispectral unmanned aerial vehicle images at various growth stages. Remote Sens. 15, 3332. doi: 10.3390/rs15133332

[B81] Zhu J. Yin Y. Lu J. Warner T. A. Xu X. Lyu M.. (2023a). The relationship between wheat yield and sun-induced chlorophyll fluorescence from continuous measurements over the growing season. Remote Sens. Environ. 298, 113791. doi: 10.1016/j.rse.2023.113791

